# Cross-Cultural Investigation of Male Gait Perception in Relation to Physical Strength and Speed

**DOI:** 10.3389/fpsyg.2017.01427

**Published:** 2017-08-21

**Authors:** Bernhard Fink, Marieke Wübker, Julia Ostner, Marina L. Butovskaya, Anna Mezentseva, José Antonio Muñoz-Reyes, Yael Sela, Todd K. Shackelford

**Affiliations:** ^1^Institute of Psychology, University of Göttingen Göttingen, Germany; ^2^Leibniz ScienceCampus Primate Cognition Göttingen, Germany; ^3^Department of Behavioral Ecology, University of Göttingen Göttingen, Germany; ^4^Research Group Social Evolution in Primates, German Primate Center Göttingen, Germany; ^5^Social Anthropology Research and Education Center, Russian State University for the Humanities Moscow, Russia; ^6^Institute of Ethnology and Anthropology, Russian Academy of Sciences Moscow, Russia; ^7^Centro de Estudios Avanzados, Universidad de Playa Ancha Valparaíso, Chile; ^8^Department of Psychology, Oakland University, Rochester MI, United States

**Keywords:** physical strength, handgrip, gait, attractiveness, male

## Abstract

Previous research documents that men and women can accurately judge male physical strength from gait, but also that the sexes differ in attractiveness judgments of strong and weak male walkers. Women’s (but not men’s) attractiveness assessments of strong male walkers are higher than for weak male walkers. Here, we extend this research to assessments of strong and weak male walkers in Chile, Germany, and Russia. Men and women judged videos of virtual characters, animated with the walk movements of motion-captured men, on strength and attractiveness. In two countries (Germany and Russia), these videos were additionally presented at 70% (slower) and 130% (faster) of their original speed. Stronger walkers were judged to be stronger and more attractive than weak walkers, and this effect was independent of country (but not sex). Women tended to provide higher attractiveness judgments to strong walkers, and men tended to provide higher attractiveness judgments to weak walkers. In addition, German and Russian participants rated strong walkers most attractive at slow and fast speed. Thus, across countries men and women can assess male strength from gait, although they tended to differ in attractiveness assessments of strong and weak male walkers. Attractiveness assessments of male gait may be influenced by society-specific emphasis on male physical strength.

## Introduction

Physical strength is a sexually dimorphic, heritable trait (Lassek and Gaulin, 2009; Isen et al., 2014) with men, on average, stronger than women. Male strength is positively related to measures of health (Fredericksen et al., 2002; Vaara et al., 2012) and sexual behavior (Gallup et al., 2007), and negatively related to mortality (Rantanen et al., 2000; Metter et al., 2002). Thus, physical strength may indicate male quality and may be used in assessments of male competitiveness and mate quality (Frederick and Haselton, 2007; Sell et al., 2009, 2010, 2012).

Previous research documents that men and women can accurately assess male physical strength from men’s faces, bodies, and voices (Sell et al., 2009, 2010), and that women prefer the dance movements of physically strong men to those of physically weak men (Hugill et al., 2009; Weege et al., 2015b). A biomechanical analysis showed that stronger men who displayed larger, more variable, and faster movements with their arms were judged as better dancers by both sexes (McCarty et al., 2013). More recently, Fink et al. (2016) reported in a German sample that both sexes judged the gait of strong men higher in strength, dominance, and attractiveness than the gait of weak men. Fink et al. (2016) documented a sex difference in attractiveness assessments of strong and weak male walkers such that women but not men judged strong walkers as more attractive than weak walkers. Thus, both sexes may use physical strength information from gait cues in assessments of male quality and competiveness.

In the present study, we expand on these findings by considering men’s and women’s perceptions of male gait in Chile, Germany, and Russia – three countries that differ in social and cultural norms (among other measures), and along indices of development (United Nations Development Programme^1^). There is little information available about how gait is perceived in different countries, and most investigations of gait are clinical or sports science studies. In a preliminary study on Brazilian and German women’s attractiveness perceptions of (British) men’s dance movements, Fink et al. (2014) reported a positive correlation of Brazilian with German women’s assessments of men’s dance attractiveness, suggesting cross-cultural similarity in dance attractiveness perceptions. Additional analysis revealed a significant difference between Brazilian and German women’s ratings of men’s dance movements, which were attributable to personality of the dancer. Thus, although there is some cross-cultural consensus in women’s perceptions of men’s dance movements, part of the variation in dance movement perception is attributable to culture-dependent personality cues derived from dance movements.

Here, we consider gait of healthy young men and investigate men’s and women’s strength and attractiveness assessments of strong and weak male walkers. In addition to strength variation in male walkers, we manipulate walking speed. Schmitt and Atzwanger (1995) reported a positive correlation of walking speed with socioeconomic status in men. Whether an effect of walking speed on perception exists, especially in a sample that is relatively homogeneous in socioeconomic status, is yet unclear. There is mixed opinion about the possible effect of gait speed on perception. If Schmitt and Atzwanger (1995) are correct, manipulating gait speed should have an effect on perception, with more positive assessments provided to fast walkers and less positive assessments provided to slow walkers. However, it is also plausible that faster movements are associated with less positive assessments, as has been reported for dance (Weege et al., 2015a), and that slow movements indicate self-composure and security, for example.

We hypothesized differences in strength perceptions of strong and weak male walkers, for both men and women, and independent of country. If such differences are observed across cultures, this would suggest a universal capacity to derive perceptions of physical strength from male gait. Following previous reports (Fink et al., 2016), we expected to detect a sex difference in attractiveness perceptions of male gait, with women providing more positive assessments to strong walkers and less positive assessments to weak male walkers, compared to men’s assessments. With regard to an effect of speed, the study was exploratory, given the lack of evidence from previous studies.

## Materials and Methods

### Gait Recordings and Manipulations

Gait recordings were obtained from 80 men, aged 18 to 42 years, recruited at Northumbria University (United Kingdom) as part of a large-scale study on body movement in relation to anthropometry and personality (for related reports, see Fink et al., 2012, 2014, 2016; Weege et al., 2012, 2015a,b; Hufschmidt et al., 2015). No participants reported any injuries that might influence natural movements.

Walk movements were recorded with an optical motion-capture system (Vicon, Oxford, United Kingdom) running Vicon Nexus software. Thirty-nine reflective markers were attached to each participant’s major joints and body parts (Plug-in-gait marker set). Participants did not receive instructions on how to walk, but were told to remain within a certain area (marked with adhesive tape on the floor) in a room dedicated to motion-capturing. A male and a female investigator was present during recordings. Gait recordings were applied to size- and shape-standardized, sex-neutral humanoid characters using Motionbuilder software (Autodesk Inc., San Rafael, CA, United States) and rendered as 773 pixel × 632 pixel video clips. A sequence of 3 s (4–5 strides) was digitally isolated from the middle of each walk sequence.

Participants handgrip strength (HGS; kgf) was measured with a hand dynamometer (Takei Kiki Kogyo K.K., Japan), twice for each hand, and the means of the two left and two right HGS measurements were used for assignment to either the “strong” or “weak” walker group. For the selection of strong and weak walkers, we considered only participants for which no issues in post-processing of walk movements were noted (e.g., gaps in the motion stream that could not be filled). Of the remaining 70 men (aged 18 to 42 years, *M* = 21.6, *SD* = 4.1), the videos of the 10 strongest (*M*_HGS_ = 48.6, *SD* = 3.2) and 10 weakest participants (*M*_HGS_ = 23.8, *SD* = 4.0) (each heterosexual, by self-report) were selected for the rating study. Strong and weak walkers differed in HGS but not in the number of strides displayed in the videos [HGS: *t*(18) = 15.20, *p* < 0.001; strides: *z* = -0.89, *p* = 0.37].

In addition to the videos displaying strong and weak male walkers at “normal” speed, two further sets of these walkers were created by manipulating playback speed, thus rendering the gait “slower” and “faster.” Slow and fast versions were created by setting playback speed to 70% (slow) and 130% (fast) of the original video using iMovie software (Apple Inc., Cupertino, CA, United States). To test for artifacts in manipulations, 15 German men and women (aged 20 to 34 years, *M* = 26.2, *SD* = 4.2) judged slow, normal, and fast versions of the videos for “naturalness” on a 4-point Likert-type scale (1 = *natural*, 4 = *unnatural*). Naturalness judgments did not differ across conditions [slow: *M* = 2.5, *SD* = 0.3, normal: *M* = 2.5, *SD* = 0.2, fast: *M* = 2.5, *SD* = 0.2; *F*(2,14) = 0.01, *p* = 0.99].

For presentation in the main experiment, three repetitions of each participant’s walk sequence (in slow, normal, and fast condition, respectively) were used to construct a new video showing walk movements in a loop.

### Gait Ratings

The gait videos were shown to 188 men and 199 women (aged 16 to 50 years), recruited at the University of Göttingen (Göttingen, Germany, *n* = 122; 60 males, *M* = 23.0, *SD* = 3.4; 62 females, *M* = 24.4, *SD* = 3.7), the Universidad de Playa Ancha (Valparaiso, Chile, *n* = 85; 46 males, *M* = 31.6, *SD* = 8.3; 39 females *M* = 31.7, *SD* = 8.8) and the Russian State University for the Humanities (Moscow, Russia, *n* = 180; 82 males, *M* = 21.5, *SD* = 3.1; 98 females, *M* = 21.5, *SD* = 4.0).

Participants judged the walk movements for strength and attractiveness using a 7-point scale (1 = *low on attribute*, 7 = *high on attribute*). Gait assessments at normal speed were collected in Chile, Germany, and Russia. The total sample size for country comparisons of strong and weak walkers at normal speed was *n* = 213. Additional assessments of strength and attractiveness for walking speed manipulations were collected in Germany (fast walkers *n* = 41, slow walkers *n* = 40) and Russia (fast walkers *n* = 51, slow walkers *n* = 42). The total sample size for country comparisons of strong and weak walkers varying in gait speed was *n* = 302.

In Germany, video ratings were collected on laptop computers running Medialab v2012 (Empirisoft Corp., New York, NY, United States) software. In Chile and Russia, videos were presented on PC monitors using SurveyMonkey^2^. The videos were embedded in the presentation via a (private) YouTube account with advertisements removed and could be replayed. Judgments of attributes were made in blocks and the order of clips was randomized within each block, with blocks randomized across participants.

## Results

**FIGURE 1:**
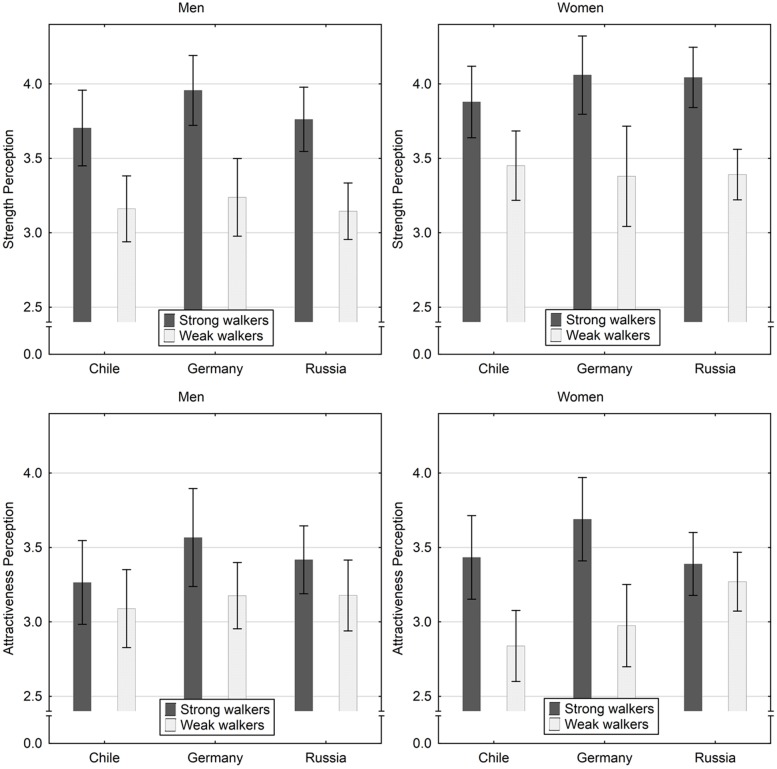
Strength and attractiveness perception (*M* and 95% CI) of strong and weak male walkers at normal speed across countries.

**Figure 1** presents descriptive statistics for men’s and women’s strength and attractiveness perceptions of strong and weak male walkers in Chile, Germany, and Russia. A mixed-design ANOVA was performed on perception measures with walker strength as a within-subjects factor and country and observer’s sex as between-subjects factors (**Table 1**). For this analysis, we considered only judgments of walkers in the “normal speed” condition. There were main effects of walker strength on perceptions of strength and attractiveness. Strong walkers were rated higher on both attributes (**Figure 1**). The interaction effect of walker strength × country was significant for perceptions of attractiveness but not strength. Gait attractiveness ratings of strong walkers were highest in Germany, followed by Russia and Chile; for weak walkers, Russian attractiveness ratings were highest, followed by German and Chilean assessments (**Figure 1**). However, *post hoc* multiple tests did not reveal significant differences for these comparisons (Tukey’s HSD test, all *p* > 0.06).

**Table 1:** Statistical comparisons of strength and attractiveness perceptions of strong vs. weak male walkers at normal speed in Chile, Germany, and Russia.

Factor	Attribute	*F*	*p*	$\eta_{p}^{2}$
Walker strength	Strength	203.98^1^	<0.001^∗∗∗^	0.50
Walker strength	Attractiveness	65.31^1^	<0.001^∗∗∗^	0.24
Walker strength × Country	Strength	2.33^2^	0.10	0.02
Walker strength × Country	Attractiveness	5.31^2^	<0.01^∗∗^	0.05
Walker strength × Sex	Strength	0.22^1^	0.64	0.001
Walker strength x Sex	Attractiveness	5.06^1^	<0.05^∗^	0.02
Walker strength × Country × Sex	Strength	0.36^2^	0.70	0.003
Walker strength × Country × Sex	Attractiveness	4.22^2^	<0.05^∗^	0.04


*^1^*F*(1,207), ^2^*F*(2,207).*

An interaction effect of walker strength × sex was detected for perceptions of attractiveness but not for strength. Women judged strong walkers higher on attractiveness than did men, and men judged weak walkers higher on attractiveness than did women (**Figure 1**), although these differences were not statistically significant [strong walkers, *t*(211) = 0.75, *p* = 0.46; weak walkers, *t*(211) = -0.83, *p* = 0.41]. Both sexes gave higher strength ratings to strong walkers as compared to weak walkers [men, *t*(106) = 11.91, *p* < 0.001; women, *t*(105) = 9.28, *p* < 0.001]. There was a three-way interaction effect of walker strength × country × sex for perceptions of attractiveness but not strength (**Table 1**). In Chile and Germany, women’s attractiveness judgments of strong male walkers were higher than those of men whereas men judged weak walkers more positively than did women. In Russia, men gave lower attractiveness scores to weak walkers as compared to women (**Figure 1**). However, paired *t*-tests did not reveal significant differences for these comparisons (all *p* > 0.16).

**Table 2** reports descriptive statistics of strength and attractiveness assessments of strong and weak male walkers at slow, normal and fast speed in Germany and Russia. To test for effects of walking speed on perceptions of strength and attractiveness of strong and weak male walkers, mixed-design ANOVAs were conducted with walker strength as a within-subjects factor and gender, playback speed, and country as between-subjects factors (**Table 3**). There were no interaction effects of walker strength × walking speed on perceptions of strength and attractiveness. However, the interaction effect of walker strength × walking speed × country was significant for perceptions of attractiveness but not strength. German and Russian participants rated strong walkers higher on attractiveness than weak walkers when viewed at slow and fast speed. At normal speed, German attractiveness ratings of strong walkers were higher than those of Russians, and for weak walkers, Russian attractiveness ratings were higher than those of Germans. However, *post hoc* multiple tests did not reveal significant differences for these comparisons (Tukey’s HSD test, all *p* > 0.11) except for German attractiveness perceptions of weak walkers at normal vs. slow speed (mean difference -0.37, *p* < 0.05). No significant 4-way interaction effect of walker strength × walking speed × country × sex was detected for perceptions of strength and attractiveness.

**Table 2:** Descriptive statistics (M ± SD) of strength and attractiveness assessments of strong and weak male walkers at slow, normal, and fast speed in Germany and Russia.

	Strong walkers			Weak walkers		
		
	Slow	Normal	Fast	Slow	Normal	Fast
**Germany**						
Strength	4.04 ± 0.57	4.00 ± 0.53	3.92 ± 0.63	3.61 ± 0.59	3.31 ± 0.64	3.47 ± 0.56
Attractiveness	3.69 ± 0.61	3.63 ± 0.66	3.55 ± 0.56	3.45 ± 0.71	3.08 ± 0.54	3.36 ± 0.67
**Russia**						
Strength	4.00 ± 0.59	3.91 ± 0.69	3.68 ± 0.66	3.33 ± 0.80	3.29 ± 0.59	3.09 ± 0.69
Attractiveness	3.53 ± 0.64	3.40 ± 0.71	3.40 ± 0.73	3.35 ± 0.78	3.23 ± 0.70	3.04 ± 0.74


**Table 3:** Statistical comparisons of strength and attractiveness perceptions of strong vs. weak male walkers at slow speed, normal speed, and fast speed in Germany and Russia.

Factor	Attribute	*F*	*p*	$\eta_{p}^{2}$
Walker strength	Strength	325.61^1^	<0.001^∗∗∗^	0.53
Walker strength	Attractiveness	53.79^1^	<0.001^∗∗∗^	0.16
Walker strength × Country	Strength	2.66^1^	0.10	0.009
Walker strength × Country	Attractiveness	1.46^1^	0.23	0.005
Walker strength × Sex	Strength	0.19^1^	0.66	0.001
Walker strength × Sex	Attractiveness	2.81^1^	0.09	0.01
Walker strength × Speed	Strength	2.00^2^	0.14	0.01
Walker strength × Speed	Attractiveness	1.64^2^	0.20	0.01
Walker strength Country × Sex	Strength	0.02^1^	0.89	0.0001
Walker strength Country × Sex	Attractiveness	0.52^1^	0.47	0.002
Walker strength Country × Speed	Strength	1.98^2^	0.14	0.01
Walker strength Country × Speed	Attractiveness	4.46^2^	<0.05^∗^	0.03
Walker strength Speed × Sex	Strength	0.51^2^	0.60	0.004
Walker strength Speed × Sex	Attractiveness	1.92^2^	0.15	0.01
Walker strength Country × Speed × Sex	Strength	0.16^2^	0.85	0.001
Walker strength Country × Speed × Sex	Attractiveness	1.36^2^	0.26	0.009


*^1^*F*(1,290), ^2^*F*(2,290).*

## Discussion

The results of the present study confirm the previously reported effect of male physical strength on perceptions of strength and attractiveness from gait (Fink et al., 2016) in three countries – Chile, Germany, and Russia. Moreover, and in accord with Fink et al. (2016) we identified an interaction of walker strength with sex of perceiver for attractiveness perceptions, but not for assessments of strength, such that women tended to provide higher judgments than men to strong walkers, and men tended to provide higher judgments than women to weak walkers. These results suggest that humans are sensitive to gait cues in order to infer male physical strength independent of perceiver’s sex and country. Further, women more than men seem to discriminate between strong and weak walkers when assessing attractiveness. These results are consistent with greater variability in women’s assessments of male attractiveness compared to men’s assessments of female attractiveness—especially when women consider a man’s status (e.g., Townsend and Wasserman, 1997). Physically stronger men may signal competitiveness in intrasexual conflict, in addition to security, self-confidence, and status, thus providing information that women across countries – at least in samples of industrialized societies – are sensitive to, and show a preference for.

Perceptions of strength distinguished between strong and weak male walkers in all three countries. Perceptions of attractiveness varied between countries and sex, although effect sizes for these interactions were small. Thus, interpretations of country- and sex-specific assessments of male gait attractiveness must be made with caution. In Chile and Germany, women judge strong male walkers to be more attractive than weak male walkers, whereas in Russia there was no such difference. In the current study, we focused on comparisons of men’s and women’s perceptions of strength from gait across countries. Thus, we did not include specific measures of, for example, social dominance orientation (SDO; Pratto et al., 1994) and other measures of individual differences in personality, which may explain further sex- and country-specific effects of strength attributions, as it was reported to be the case for women’s perceptions of men’s dances (Fink et al., 2014).

Manipulations of playback speed of gait did not affect strength perceptions of men or women in either country in which they were investigated (Germany and Russia). Thus, the assessment of physical strength from gait cues appears to be independent of gait speed. German and Russian participants judged strong walkers as more attractive when viewed at slow and fast speed. Germans tended to judge strong walkers higher on attractiveness than Russians when viewed at normal speed and Russian attractiveness judgments of weak walkers were higher than those of Germans. Again, because of the small effect sizes, these results should be interpreted with caution. The manipulation of playback speed of gait videos should be regarded as approximation to “ideal” stimuli, thus providing only preliminary information about perceptions of gait speed. Walking faster or slower is associated with changes in movement biomechanics, and such changes were not considered in our manipulations. Future research may profitably investigate whether it is, in fact, gait speed changes that influence women’s attractiveness perceptions of male walkers (as suggested by the current study), or if altering gait speed is linked to other gait characteristics that affect perception.

## Conclusion

We demonstrate an effect of male gait on perceived strength in three countries, suggesting that the capacity to derive physical strength information from male gait is not culture-specific. Women more than men seem to discriminate between strong and weak walkers when assessing attractiveness of male gait. Attractiveness assessments of male gait show some local variation, and may be influenced by society-specific emphasis of male physical strength. Future studies investigating the effect of strength from gait cues on perception should test whether the reported effects extend to pre-industrialized societies. If this is the case, it would support the suggestion that specific biomechanical characteristics of male gait are linked to physical strength, and that humans may be adapted to use these cues in social perception.

## Ethics Statement

This study was carried out in accordance with the recommendations of the German Psychological Society. All subjects gave written informed consent in accordance with the Declaration of Helsinki. In case of minors, consent was additionally obtained from their legal guardians or teachers. The protocol was approved by the ethics committees of the University of Göttingen and Northumbria University.

## Author Contributions

BF, MW, and JO conceived the study. MW, MB, AM, and JM-R collected data. BF and MW analyzed the data. BF, JO, MB, YS, and TS wrote the manuscript. All authors edited the manuscript for intellectual content, provided critical comments on the manuscript, and agreed to be accountable for the content of the work.

## Conflict of Interest Statement

The authors declare that the research was conducted in the absence of any commercial or financial relationships that could be construed as a potential conflict of interest.

## Footnotes

1 http://hdr.undp.org/en

2 www.surveymonkey.com
